# Abdominal only CPR during cardiac arrest for a patient with an LVAD during resternotomy: A case report

**DOI:** 10.1186/1749-8090-6-91

**Published:** 2011-07-15

**Authors:** Eric M Rottenberg, Jarrett Heard, Robert Hamlin, Benjamin C Sun, Hamdy Awad

**Affiliations:** 1 301B Fenway Road, Columbus, OH, 43214, USA; 2 The Ohio State University College of Medicine, 370 West 9th Avenue, Columbus, OH, 43210, USA; 3 The Ohio State University College of Veterinary Medicine, 1900 Coffey Road, Columbus, OH, 43210, USA; 4 The Ohio State University Medical Center, Department of Surgery, Division of Cardiothoracic Surgery, N847 Doan Hall, 410 West 10th Avenue, Columbus, OH, 43210, USA; 5 The Ohio State University Medical Center, Department of Anesthesiology, N411 Doan Hall, 410 West 10th Avenue, Columbus, OH, 43210, USA

## Abstract

We present a case in which a patient with a previous sternotomy and left ventricular assist device (LVAD) implantation developed cardiac arrest during resternotomy for LVAD exchange. The surgeon refused chest compressions for fear of potential damage to the inflow cannula directly beneath the sternum. The perioperative team had no alternatives to external cardiac massage other than rapid deployment of extra-corporeal membrane oxygenation for mechanical support, so the anesthesiologist advised the nursing personnel to perform abdominal only cardiopulmonary resuscitation while the surgeon performed a femoral bypass to cannulate the groin for extra-corporeal membrane oxygenation support.

## Background

Cardiac arrest during cardiac surgery is a unique situation. In 2009, the European Society of Cardiothoracic Surgery published a separate guideline that addressed these particular situations, including the timing of emergency resternotomy, the number of attempts at defibrillation before reopening, the administration of epinephrine, and emergency resternotomy sets [1]. However, this guideline did not address the treatment of patients with a mechanical assist device in cardiac arrest situations since their treatment is highly complicated. Cardiac arrest may be due to mechanical failure and external cardiac massage (ECM) is not appropriate, as stated by the European Society of Cardiothoracic Surgery [1].

We present a case in which the patient with a previous sternotomy and LVAD (HeartMate II, Thoratec Corporation) implantation developed cardiac arrest during resternotomy for LVAD exchange due to hemolysis. The surgeon denied chest compressions for fear of potential damage of the inflow cannula directly beneath the sternum. As there were no alternatives to ECM offered by the American Heart Association and the European Society of Cardiothoracic Surgery [1,2] other than rapid deployment of extra-corporeal membrane oxygenation (ECMO) for mechanical support, we performed abdominal only cardiopulmonary resuscitation (AO-CPR) while the surgeon performed a femoral bypass to cannulate the groin for ECMO support.

## Case Presentation

A 56-year-old male with multiple co-morbidities, including a long-standing history of non-ischemic dilated cardiomyopathy, stage III chronic kidney disease, and congestive hepatopathy, underwent LVAD implantation two months prior to the most recent admission. He returned to the hospital due to persistent atrial fibrillation, progressively worsening dyspnea on exertion and rest, abdominal distension with ascites, and suspected ongoing hemolysis due to positioning of the inflow cannula at the apex of the LVAD.

It was decided that the patient should return to the operating room for placement of a new LVAD due to hemolysis and hypotension refractory to medical management. The night before the scheduled surgery, the patient was intubated due to worsening cardiopulmonary parameters, including increased work to breathe, and maintained on epinephrine 0.15 mcg/kg/min, norepinephrine 0.1 mcg/kg/min and dobutamine 3 mcg/kg/min. He was transferred to the operating room to replace the pump. Pre-op vitals included: temp 37.6 degrees Celsius, arterial blood pressure 64/50, mean arterial pressure 55, heart rate 118 and respiratory rate of 16. Prior to induction of anesthesia, labs included: white blood cells 15.2, hemoglobin 10, hematocrit 29.4, platelets 96, Na+ 130, K+ 3.1, Cl- 95, CO_2 _25, blood urea nitrogen 24, creatine 2.08, glucose 84, and international normalized ratio 3.5. Preoperative arterial blood gases were pH 7.48, pCO_2 _35.9, pO_2 _184.2, and HCO_3 _26.1. In the operating room, hemodynamic parameters were continuously monitored via radial arterial line and Swan-Ganz catheter. Induction was uneventful with etomidate 10 mg and cisatracurium 10 mg. The transesophageal echocardiography (TEE) probe was placed uneventfully. The surgeons entered the mediastinum using the previous sternal incision. Once they began dissecting out the mediastinum, the patient became severely hypotensive and asystolic, and the TEE did not detect any movement on both the left and right side of the heart.

**Figure 1:**
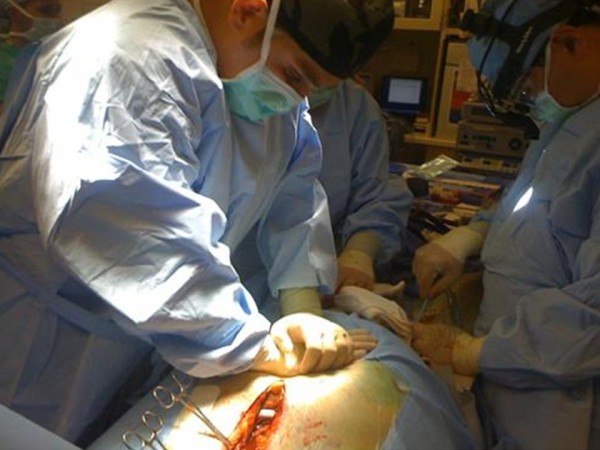
**Abdominal only cardiopulmonary resuscitation during cardiac arrest in patient with HeartMate**. Abdominal only cardiopulmonary resuscitation using a left paramedian technique 1 to 2 inches left of the midline while the surgeon performs cannulation of the femoral artery and vein for placement of extra-corporeal membrane oxygenation for long-term mechanical support.

**Figure 2:**
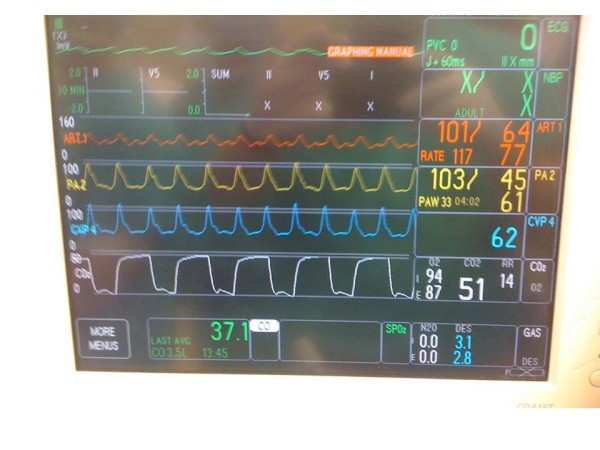
**Monitor after 15 minutes of abdominal only cardiopulmonary resuscitation**. Monitor showing the hemodynamic waveforms and their means during abdominal only cardiopulmonary resuscitation during cardiac arrest while the surgeon performed cannulation of the femoral artery and vein as shown in Figure 1. Coronary perfusion pressure: Mean arterial pressure (MAP) - central venous pressure (CVP) = 15 mmHg.

The anesthesiologist alerted the surgeon that Advanced Cardiac Life Support (ACLS) protocol was needed and the surgeon communicated that chest compressions were contraindicated due to the position of the inflow cannula directly beneath the sternum. The anesthesiologist recommended AO-CPR with manual mid-abdominal compressions 1 to 2 inches left of midline (left paramedian) at a rate of 80 beats/min with maximal force while the surgeon cannulated the groin to provide long-term mechanical support in the form of ECMO. As instructed, two members of the team performed AO-CPR (Figure 1). During ACLS, the patient continued to be mechanically ventilated and epinephrine, vasopressin, and sodium bicarbonate were given per ACLS protocol, and the hemodynamic parameters as a result of AO-CPR continued to be monitored (Figure 2). The duration of the CPR was 15 minutes, during which time the surgeon was able to cannulate the femoral artery and vein and institute ECMO support. The chest was closed and the patient was transferred to the intensive care unit. The patient spent 24 hours in the intensive care unit on ECMO support and mechanical assist device. A decision was made to withdraw care after 24 hours and the patient expired.

Our case represents a difficult situation where the perioperative team faced a new challenge in the operating room: what are the alternatives to ECM when chest compressions are contraindicated due to position of the inflow cannula directly beneath the sternum? Neither the new guidelines published in the *European Journal of Cardiothoracic Surgery *in 2009 nor the American Heart Association in 2010 provided alternatives to ECM for patients with a mechanical assist device.

It became evident that there was a need for an alternative to ECM, such as AO-CPR, to protect the recent sternotomy until re-opening of the chest to provide internal cardiac massage. The *Interactive Cardiovascular Thoracic Surgery *e-community conducted a discussion to address whether AO-CPR could be used instead of ECM to either protect the recent sternotomy or while chest compressions are not possible during resternotomy [3]. After reviewing this evidence, Dunning et al. [1] concluded that AO-CPR theoretically has the potential to provide adequate systemic perfusion while an emergency resternotomy is being performed, but further evidence is needed before it can be recommended for routine use.

In general, coronary perfusion pressure during cardiac arrest is the difference between pressure in the aorta (from which the coronary arteries arise) and the right atrium (into which the coronary arteries exit). Using a mathematical model to describe the biophysics of cardiopulmonary resuscitation with periodic z-axis acceleration or abdominal compression at aortic resonant frequencies, Babbs [4] proposed that differences in wave mechanics, resulting from simultaneous compression of the abdominal aorta and the inferior vena cava, produced differences in pressure between the aorta and right atrium. During CPR, the minimal coronary perfusion pressure considered necessary for successful resuscitation with return of spontaneous circulation (ROSC) is 15 mmHg [5]. The values for mean aortic and central venous pressure for our patient were 77 and 62 mmHg, respectively, which provided a mean coronary perfusion pressure of 15 mmHg (77 to 62 mmHg). In a study of 100 patients, however, conventional CPR provided a mean CPP of only 12.5 mmHg [5]; thus, we propose that the abdominal only CPR in our patient could have served as an effective bridge between the arrest and initiation of ECMO.

In our case, AO-CPR was unplanned, but the surgeon refused chest compressions due to contraindications in this patient. Due to lack of alternatives for resuscitation other than ECMO in this patient, the anesthesiologist suggested that AO-CPR be performed as a temporary resuscitative effort until the surgeon could successfully cannulate the femoral artery and vein to provide long-term mechanical support. Two rescuers performed AO-CPR with generation of coronary perfusion pressure (CPP) of 15 mmHg for 15 minutes, the duration of resuscitation. Both achieved results that appeared to be identical. The evidence seems to suggest that AO-CPR in this particular situation may be comparable to ECM in generating adequate CPP, but at this point it is still too early to determine the true efficacy of AO-CPR compared to ECM with regards to ROSC and neurological outcome.

Other evidence of adequate CPP generated during AO-CPR includes that from Geddes and colleagues [6] and Pargett et al. [7] who compared AO-CPR with chest compressions in animal models, and showed that AO-CPR was equivalent or superior to standard chest compressions at providing coronary perfusion. Neither of these studies reported any visceral organ damage or contraindications to AO-CPR, nor did they comment on neurological outcome. At this time, we cannot comment on the efficacy of AO-CPR on neurological outcomes as neurological status could not be assessed in our patient because he was intubated, sedated and paralyzed until care was withdrawn.

This case report generates important concerns. In our patient, even though we generated a CPP of 15 mmHg, which has been shown in some patients to be adequate, we do not know whether it was adequate to allow ROSC or whether the ECMO was responsible for ROSC. Other concerns are: what is the optimal delivery (optimal rate, depth/force, duty cycle and location of hand position) of AO-CPR in achieving successful resuscitation with ROSC, what is the best strategy for ventilation during AO-CPR, and is there potential damage/injury to abdominal viscera during AO-CPR?

## Conclusions

In conclusion, this case demonstrated an example where, due to contraindications to ECM in a patient with a mechanical assist device, we were able to successfully provide an alternative means of CPR. This alternative technique was done with no delay and without creating wound dehiscence while the surgeon was working, achieving adequate perfusion as measured by CPP, mean arterial pressure and systolic blood pressure, and providing a bridge to ECMO support. As a result, we believe that further animal and human studies need to be performed before the technique can be adopted as a valid method of resuscitation in this unique situation.

## Consent

Obtaining consent was a difficult endeavor since the patient died during his hospitalization at our institution in 2010. We contacted the Institutional Review Board (IRB) and spoke with an exempt analyst, Ms. Sherry Pettey, whose contact information is listed below, who said that we did not need IRB approval for submission of the case report. Per the request of the *Journal of Cardiothoracic Surgery*, we attempted to contact the patient's next of kin, his wife. Unfortunately, the only number listed has been disconnected and we were unable to find another listing to try and reach her. We also contacted his former place of employment to determine if it had any contact information of family or next of kin, which also could not provide us with any current contacts. As such, we believe that we performed our due diligence in getting informed consent, but due to the time lapse between the events surrounding the case and the current submission of the case report as well as the physical passing of the patient, we were unsuccessful in obtaining informed consent.

Sherry Pettey

1960 Kenny Rd

300 Research Foundation Building

Columbus, OH 43210

Pettey.6@osu.edu

614-688-0389

## Abbreviations

ACLS: advanced cardiac life support; AO-CPR: abdominal-only cardiopulmonary resuscitation; CPP: coronary perfusion pressure; CPR: cardiopulmonary resuscitation; ECM: external cardiac massage; ECMO: extra-corporeal membrane oxygenation; LVAD: left ventricular assist device; ROSC: return of spontaneous circulation; TEE: transesophageal echocardiography.

## Competing interests

The author declares that they have no competing interests.

## Authors' contributions

All authors have read and approved the final manuscript.

ER: Designed the study, conducted the study, analyzed the data, and wrote the manuscript.

JH: Analyzed the data and wrote the manuscript.

RH: Designed the study, conducted the study, and analyzed the data.

BS: Conducted the study.

HA: Designed the study, conducted the study, analyzed the data, and wrote the manuscript.
